# The Rtr1p CTD phosphatase autoregulates its mRNA through a degradation pathway involving the REX exonucleases

**DOI:** 10.1261/rna.055723.115

**Published:** 2016-04

**Authors:** Domagoj Hodko, Taylor Ward, Guillaume Chanfreau

**Affiliations:** 1 Department of Chemistry and Biochemistry, University of California, Los Angeles, Los Angeles, California 90095, USA; 2 Molecular Biology Institute, University of California, Los Angeles, Los Angeles, California 90095, USA

**Keywords:** RTR1, REX, RNA, yeast, degradation, autoregulation

## Abstract

Rtr1p is a phosphatase that impacts gene expression by modulating the phosphorylation status of the C-terminal domain of the large subunit of RNA polymerase II. Here, we show that Rtr1p is a component of a novel mRNA degradation pathway that promotes its autoregulation through turnover of its own mRNA. We show that the 3′UTR of the *RTR1* mRNA contains a *cis* element that destabilizes this mRNA. *RTR1* mRNA turnover is achieved through binding of Rtr1p to the *RTR1* mRNP in a manner that is dependent on this *cis* element. Genetic evidence shows that Rtr1p-mediated decay of the *RTR1* mRNA involves the 5′-3′ DExD/H-box RNA helicase Dhh1p and the 3′-5′ exonucleases Rex2p and Rex3p. Rtr1p and Rex3p are found associated with Dhh1p, suggesting a model for recruiting the REX exonucleases to the *RTR1* mRNA for degradation. Rtr1p-mediated decay potentially impacts additional transcripts, including the unspliced *BMH2* pre-mRNA. We propose that Rtr1p may imprint its RNA targets cotranscriptionally and determine their downstream degradation mechanism by directing these transcripts to a novel turnover pathway that involves Rtr1p, Dhh1p, and the REX family of exonucleases.

## INTRODUCTION

Messenger RNA (mRNA) degradation in eukaryotes is a critical part of gene expression control. A single mRNA in a yeast cell may produce thousands of proteins, as on average there are 4000 proteins per cognate mRNA (García-Martínez et al. 2007). Cellular mRNA concentrations are thus tightly regulated at both the level of transcription and degradation since even just one fully processed and exported mRNA molecule may drastically impact protein expression. Proteins that bind to specific *cis* regulatory elements within 3′UTRs play a major role in post-transcriptional regulatory mechanisms (for review, see Glisovic et al. 2008). These RNA binding proteins (RBPs) that bind 3′UTRs play an important role in modulating gene expression through their impact at various steps in the mRNA lifetime including mRNA processing, export, localization, turnover, and translation. Genome-wide targets of the well-characterized PUF proteins and other RBPs have been identified through the use of either affinity purification or UV crosslinking of RNA-protein complexes in vivo (Hogan et al. 2008; Freeberg et al. 2013; Wilinski et al. 2015). These studies have aided in determining the genome-wide impact of RBPs.

RBPs affecting target mRNAs through decay processes normally enhance the degradation of the target mRNA by recruitment of degradation machineries. RBPs have previously been reported to interact with factors involved in various steps in the degradation pathway. Prior to degradation by exonucleases, mRNAs must undergo deadenylation and decapping. As the first step of mRNA decay, deadenylation is first carried out by the Pan2–Pan3 complex and further digested by the Ccr4-Not complex (for review, see Norbury 2013). Some RBPs, like the PUFs, are known to activate degradation of target mRNAs through their interaction with Pop2p, a member of the Ccr4-Not complex (Goldstrohm et al. 2006; Kang et al. 2014). After deadenylation, mRNA may be degraded by the exosome or decapped by Dcp1p/Dcp2p, which are recruited by the Pat1–Lsm1–Lsm7 complex (Bouveret et al. 2000; Tharun et al. 2000; Coller and Parker 2004). In addition, the cytoplasmic DExD/H-box helicase, Dhh1p, plays a central role in linking deadenylation and decapping. Many studies have demonstrated the interaction between Dhh1p and components of the Ccr4–Not complex, Pop2p, and the Pat–Lsm1–Lsm7 complex and its role in stimulating decapping (Hata et al. 1998; Coller et al. 2001; Fischer and Weis 2002; Maillet and Collart 2002; Carroll et al. 2011; Sweet et al. 2012). Dhh1p has also been shown to interact specifically with the Rbp1p RBP (Nrg1p), to stimulate decay of the *POR1* mRNA suggesting that in some cases, Dhh1p may be a determinant in mRNA decay (Chang and Lee 2012). This is consistent with the observation that tethering Dhh1p to an mRNA is sufficient to target the mRNA for degradation (Carroll et al. 2011).

The main exonucleases recognized to degrade bulk mRNA and mRNAs targeted for decay by specific degradation pathways are the nuclear exosome, the cytoplasmic exosome, the nuclear 5′–3′ exonuclease, Rat1p, and the cytoplasmic 5′–3′ exonuclease, Xrn1p (for review, see Parker 2012). Xrn1p has been recognized as the cell's workhorse for degrading the bulk of cytoplasmic mRNA in both general and specific degradation pathways (Long and McNally 2003; van Dijk et al. 2011). 5′ capped and 3′ polyadenylated mRNAs typically undergo deadenylation-dependent decapping prior to processive 5′–3′ degradation by Xrn1p. The alternate pathway for degradation involves deadenylation and 3′–5′ degradation by the exosome. In addition to the exosome, the RNA exonuclease factors, or REXs, have homology with the RNase D type exonucleases from *E. coli* (van Hoof et al. 2000). The Rex proteins, like the exosome, are known to be involved in the 3′-end processing of ncRNAs such as snRNAs, the 5S and 5.8S rRNAs, and the RNA component of RNase MRP, but not in the degradation of mRNAs (van Hoof et al. 2000).

In addition to general degradation pathways, mRNA surveillance pathways such as the nonsense mediated mRNA decay (NMD) also regulate gene expression. NMD is initiated upon the binding of Upf1p to Sup35p at a stop codon recognized as “aberrant” (Czaplinski et al. 1998). The assembly of the other UPF factors, Upf2p and Upf3p, proceeds resulting in the rapid degradation of the transcript usually through deadenylation-dependent decapping followed by 5′–3′ degradation by Xrn1p. NMD takes place independently of Dhh1p (Coller et al. 2001; Fischer and Weis 2002).

Recent research suggests that RNA turnover is tightly connected to transcription and that mRNA degradation factors influence the rate of transcription and vice versa (Sun et al. 2012, 2013; Haimovich et al. 2013; Braun and Young 2014). Particularly, Xrn1p appears to play an important role in “buffering” mRNA levels by decreasing the transcription rate, for example, when mRNA degradation rates are slowed (Sun et al. 2013; Medina et al. 2014). Conversely, the transcriptional machinery may “imprint” a transcript with transcription factors that determine the downstream translation or decay rates (for review, see Dahan and Choder 2013).

The present study identifies a novel role for the Rtr1p transcription factor in mediating mRNA degradation. Rtr1p, (regulator of transcription 1), was previously identified as a phosphatase that dephosphorylates Ser5 and Tyr1 of the RNA polymerase II CTD tail, thus establishing a role for this protein in regulating transcription elongation and termination (Gibney et al. 2008; Mosley et al. 2009; Hsu et al. 2014). In this work we show that Rtr1p autoregulates its own mRNA post-transcriptionally and that this degradation pathway involves the 3′–5′ exonucleases Rex2p and Rex3p and the Dhh1p helicase. Rtr1p-mediated mRNA decay is a novel mRNA degradation pathway that contributes to the autoregulation of *RTR1* by its own protein product and potentially targets other transcripts like the unspliced *BMH2* transcript. We propose that Rtr1p may imprint its mRNA cotranscriptionally and determine its downstream degradation rate by targeting the transcript to this specific turnover pathway. These results identify a novel function for Rtr1p in controlling gene expression and provide evidence that mRNA decay may take place using nonclassical exonucleases.

## RESULTS

### Rtr1p autoregulates *RTR1* mRNA levels through the use of a 3′UTR *cis* element

**FIGURE 1.**
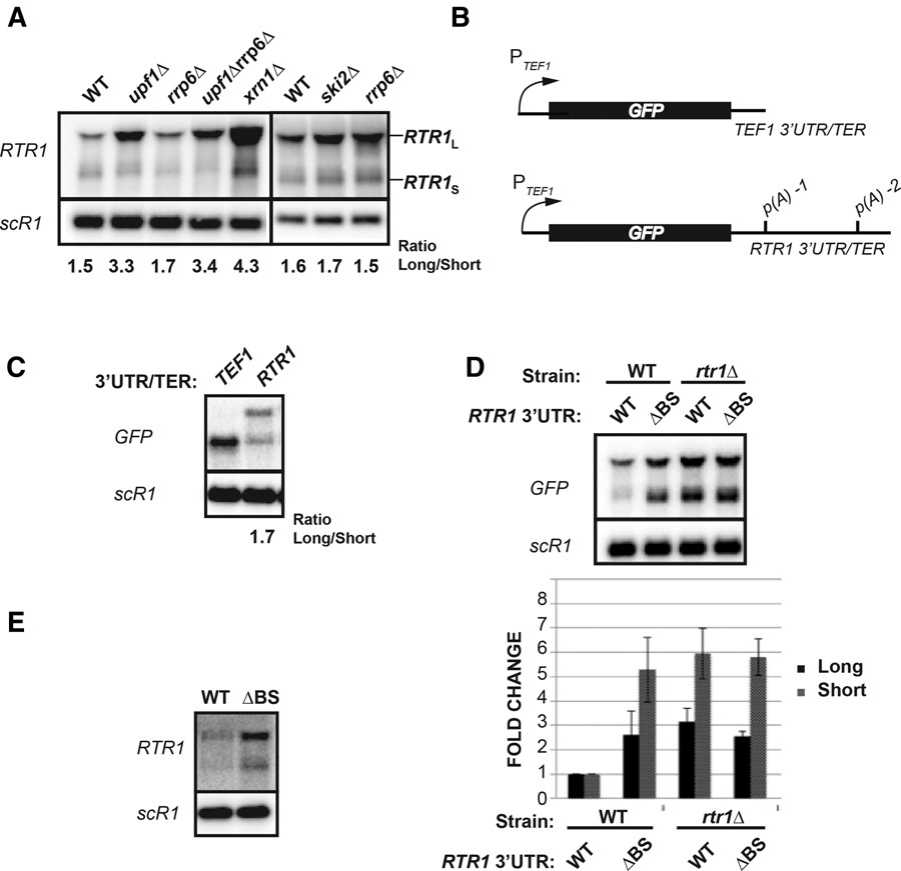
Analysis of *RTR1* mRNAs or *RTR1* 3′UTR-containing mRNAs in steady-state conditions. (*A*) Northern blot analysis of steady-state *RTR1* mRNAs in mRNA degradation mutants detected using an in vitro transcribed ^32^P-radiolabeled RNA antisense to the *RTR1* ORF (riboprobe). Cultures were grown in YPD prior to harvesting during log phase at OD 0.4–0.5. The quantitated ratio between the long and short isoform is shown *below* the blots. (*B*) Schematic representation of the GFP-TEF1_3′UTR/TER_ or the GFP-RTR1_3′UTR/TER_ cloned into the pRS404 vector. (*C*) Northern blot analysis of WT BMA64-a strains harboring the GFP-TEF1_3′UTR/TER_ or the GFP-RTR1_3′UTR/TER_ plasmids. Cultures were grown in SD-TRP, harvested at OD 0.4–0.5, and the Northern blots were probed with an in vitro transcribed ^32^P-radiolabeled RNA antisense to the *GFP* ORF. The ratio for the long and short isoform for the RTR1 3′UTR/TER transcripts is shown *below* the lane. (*D*) Northern blot analysis of WT or *rtr1*Δ cells transformed with either the GFP-RTR1_3′UTR/TER_ or *GFP-RTR1*_3′*UTR*, Δ*BS*_ plasmids. The graph *below* plots the average values of steady-state *RTR1*_*L*_ (black columns) or *RTR1*_*S*_ (gray columns) mRNAs with standard deviations resulting from three biological replicates. Relative intensity for each sample was normalized to the *scR1* loading control prior to normalizing the fold change to the WT/ GFP-RTR1_3′UTR/TER_ sample. (*E*) Steady-state analysis of the endogenous *RTR1* 3′UTR *cis* element deletion (ΔBS) as compared to the WT strain. Cultures were grown in SDC, harvested at log phase at OD 0.5, and Northern blotting was performed with the *RTR1* riboprobe.

Inspection of data obtained by previous tiling arrays and RNA-seq analysis of NMD mutants revealed an up-regulation of the *RTR1* mRNA in these mutants (Sayani et al. 2008; Kawashima et al. 2009). Our initial survey of *RTR1* mRNAs by Northern blotting and 3′ RACE revealed two major 3′ end processing isoforms, *RTR1*_L_ and *RTR1*_S_. As expected from the faux 3′UTR NMD model, which proposed that mRNAs with long 3′UTRs may be more sensitive to NMD (Amrani et al. 2004), only the longer *RTR1* mRNA isoform with a 3′UTR length of 726 nt is targeted by the NMD system (Fig. 1A), based on increased accumulation upon deletion of the NMD component Upf1p. We analyzed the expression of *RTR1* in the deletion of the nuclear exosome component, *rrp6*Δ, or the double mutant *rrp6Δupf1*Δ, because previous work showed the cooperative degradation by the NMD system and the nuclear exosome of certain unspliced mRNAs (Sayani and Chanfreau 2012); however, based solely on these steady-state analyses, *RTR1* mRNAs are targeted only by the NMD system and the nuclear and cytoplasmic exosomes both do not appear to impact *RTR1* mRNAs levels (Fig. 1A).

Due to Rtr1p's role in altering the phosphorylation status of Ser5 and Tyr1 of the RNAP II CTD heptad repeat, and given the importance of these residues in the recruitment of transcription termination factors, we explored the possibility that Rtr1p may affect its own 3′ end processing site selection. This would impact the size of the 3′UTR, and potentially the susceptibility of the *RTR1* transcripts to NMD. To test this hypothesis, we cloned the *RTR1* 3′UTR and terminator region into a plasmid (pRS404) downstream from the *GFP* ORF expressed from the *TEF1* promoter (Fig. 1B). This construct, *GFP-RTR1*_3′*UTR/TER*_, successfully expresses the *GFP* mRNA with two 3′UTR isoforms, recapitulating the *RTR1* isoforms expressed from the endogenous locus with a very similar ratio between long and short isoform abundance (compare ratios in Fig.1A,C). Surprisingly, expressing the *GFP-RTR1*_3′*UTR*_ mRNA in the *rtr1*Δ strain resulted in an increase in the steady-state abundance of both *RTR1*_S_ (approximately fivefold) and *RTR1*_L_ (approximately threefold) in comparison to the wild-type strain (Fig. 1D). Since deletion of Rtr1p affected the overall abundance of both isoforms, we hypothesized that there may be a feature present in the 3′UTR affecting the overall expression or stability of these transcripts. Previous gPAR-CLIP data (Freeberg et al. 2013) revealed a crosslinking site for cellular RNA binding proteins in the UAAUUCAUCAUCAUA sequence located 64 to 78 residues downstream from the stop codon within the 3′UTR of *RTR1*. We thus examined the effect of deleting this potential binding site (BS) to determine whether RNA binding proteins (RBPs) may affect the post-transcriptional stability of the *RTR1* mRNAs through NMD or another pathway. Strikingly, deletion of the UAAUUCAUCAUCAUA sequence in the 3′UTR (ΔBS) resulted in an increase in *RTR1* isoform levels that was comparable to the increase observed in the *rtr1*Δ strain (Fig. 1D). Moreover, no further increase in the abundance of these forms was detected in the ΔBS construct expressed in the *rtr1*Δ strain, suggesting that the regulation of *RTR1* mRNA levels through the RBP site depends on Rtr1p. This effect of the 3′UTR binding site on *RTR1* expression was also detected on the endogenous *RTR1* locus as we found that deletion of the binding site (ΔBS) within the 3′UTR of the chromosomal *RTR1* locus using the delitto perfetto approach (Storici and Resnick 2006) resulted in an increase in the overall abundance of the *RTR1* mRNAs expressed from the endogenous locus (Fig. 1E).

**FIGURE 2.**
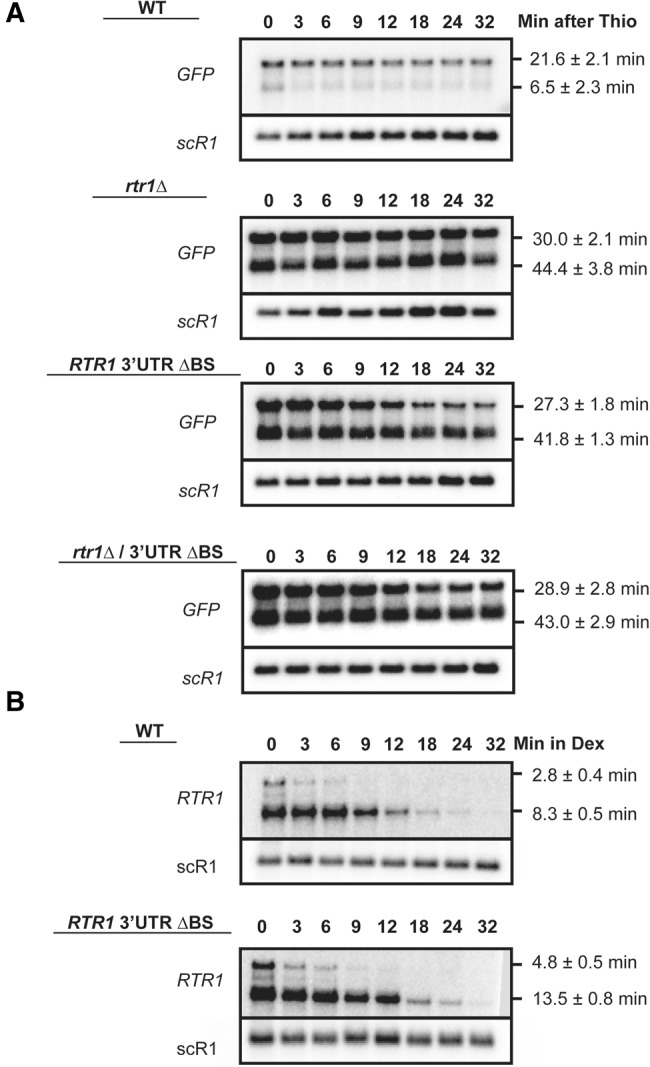
Transcription shut-off analysis of plasmid-borne or endogenous mRNAs. All calculated half-lives are the average of three independent biological replicates with standard deviation and shown at the *right* side of the blot. (*A*) Post-transcriptional stability of the *RTR1* containing mRNAs determined by addition of 3 μg/mL Thiolutin during the log phase, OD 0.5. The WT or *rtr1*Δ strains were used with either the WT *RTR1* 3′UTR/TER plasmid or ΔBS *RTR1* 3′UTR/TER. Time points were harvested at the indicated times. (*B*) The GAL1 promoter was integrated into the *RTR1* locus upstream of the ORF in either the WT or ΔBS strains. Post-transcriptional stability of the endogenous *RTR1* transcripts expressed from the GAL1 promoter was subsequently determined by shifting the cultures from 2% galactose to 4% dextrose and harvesting samples at the indicated time points.

To rule out that the changes in mRNA levels were due to changes in transcriptional output as a result of the absence of Rtr1p, we measured mRNA stability through the use of the transcriptional inhibitor, thiolutin. In these experiments, a large increase in half-life was observed for the *GFP-RTR1*_S_ mRNA when *RTR1*, the 3′UTR binding site, or both the binding site and *RTR1* are deleted, while a more modest increase in half-life is observed for the *GFP-RTR1*_L_ mRNA (Fig. 2A). Since the long *RTR1* transcript is targeted for decay by the NMD pathway (Fig. 1A), it is likely that the NMD pathway largely degrades the long transcript in the absence of the decay pathway mediated by Rtr1p or the binding site element, explaining the more modest effect of these mutations. As described previously with steady-state assays, the effect of deleting *RTR1* appears to be epistatic to deleting the binding site within the plasmid-borne mRNA. This result provides genetic evidence that Rtr1p participates in the auto-regulation of its own mRNAs via modulation of post-transcriptional stability of *RTR1* through its 3′UTR sequence. The effect of the 3′UTR element on *RTR1* stability was also detected on *RTR1* transcripts expressed from the endogenous locus. Though changing the promoter can potentially alter mRNA stability (Trcek et al. 2011), we utilized a galactose-driven promoter to shut off transcription of the *RTR1* chromosomal copy. We detected an increase in the half-life of both the long and short *RTR1* isoforms in the ΔBS mutant as compared to the wild-type 3′UTR (Fig. 2B). We note that the half-life values obtained with the thiolutin experiment and with the GAL promoter system are different. This could be due to differences in the kinetics of transcriptional shutoff between the GAL promoter and the Thiolutin treatment. In addition, Thiolutin could have indirect effects that could impact RNA half-lives (Pelechano and Pérez-Ortín 2008); additionally, differences in promoters may also impact RNA half-life (Trcek et al. 2011). However, the effects of the different mutations are the same regardless of the system used to measure *RTR1* decay: Inactivation of Rtr1p and deletion of the binding site element stabilize *RTR1*, and inactivation of both does not result in an increase in stability.

**FIGURE 3.**
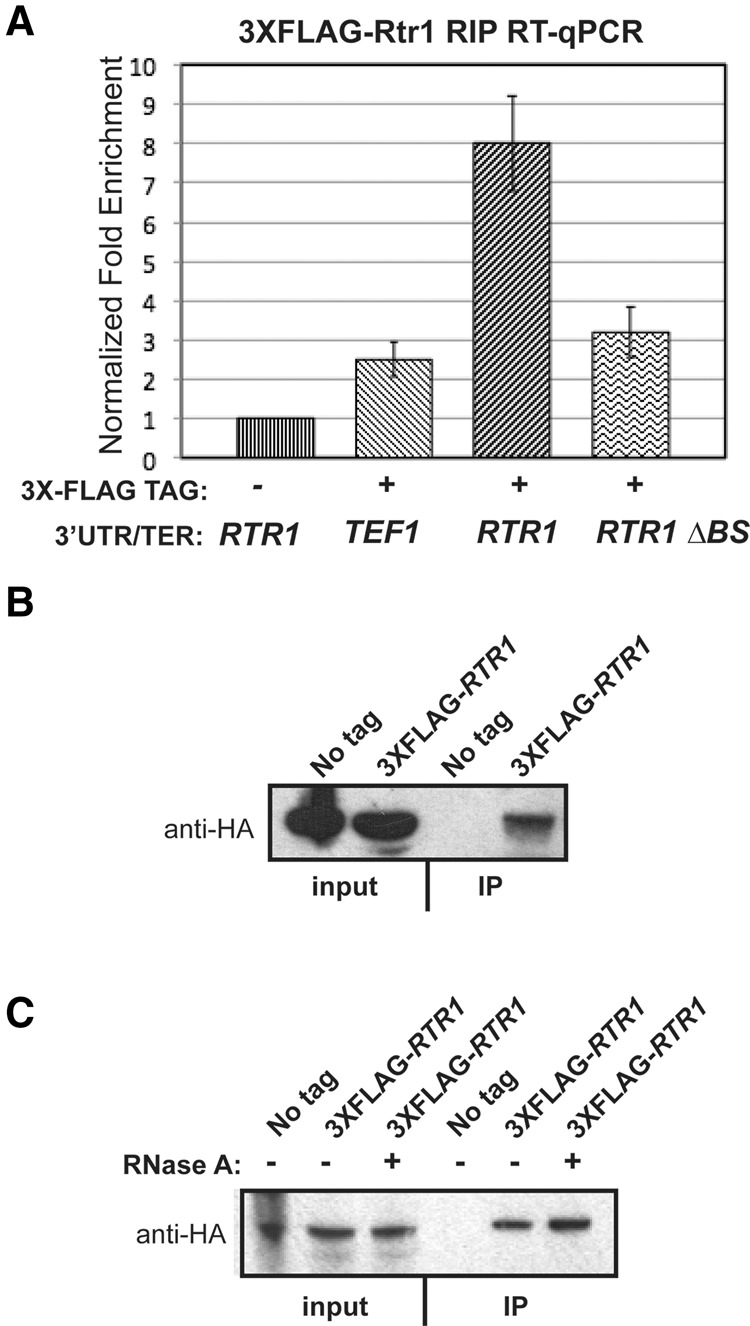
Association of 3X-Flag tagged Rtr1p with the *RTR1* 3′UTR –containing *mRNP* complex and tagged Dhh1p. (*A*) RNA immunoprecipitation (RIP) assay performed using the endogenously tagged 3X-Flag-*RTR1* strain. Cultures were grown in SD-TRP to maintain either the GFP-TEF1_3′UTR/TER_, GFP-RTR1_3′UTR/TER_, or *GFP-RTR1*_3′*UTR*, Δ*BS*_ plasmids. A qPCR was performed on the reverse-transcribed RNA input and IP samples with the GFP-FAM Taqman assay. A WT strain with the WT GFP-RTR1_3′UTR/TER_ plasmid was used as a negative control and a fold enrichment from this negative control was calculated for all other samples. All samples were normalized to the input Ct. (*B*) Coimmunoprecipitation assay performed utilizing the 3X-Flag-*RTR1* strain and the tagged Dhh1p expressed from the BG1805 vector (Yeast ORF collection from Dharmacon). Lysed samples were immunoprecipitated with protein A sepharose beads in the presence of Protease 3C. Western blotting was performed with the anti-HA primary antibody to detect the tagged Dhh1. (*C*) Co-IP assay performed the same as in *B*, except, RNase A was added into the lysate in the indicated samples.

Because gPAR-CLIP unambiguously defines crosslinking sites of all RBPs genome-wide (Freeberg et al. 2013), we aimed to identify the RBP that contributes to degradation of the *RTR1* mRNAs by binding to this 3′UTR element. Since the sequence of the *RTR1* 3′UTR BS closely resembles the consensus element for Puf1p and Puf2p binding sites, we tested the deletion of the individual *PUF* genes, *puf1Δ puf2*Δ, as well as a deletion mutant of five *PUF* genes, *5Δ pufs (*Hogan et al. 2008)*.* None of these mutants showed substantial changes in steady-state mRNA abundance as compared to the isogenic WT strain (Supplemental Fig. 1A). Additionally, deletion mutants of several other characterized RBPs, including *rbp1*Δ (*nrg1*Δ)*,* likewise had no effect on the steady-state abundance of *RTR1* mRNAs (Supplemental Fig. 1B). Based on these results and on the epistatic effects of the *RTR1* and 3′UTR element deletions, we hypothesized that Rtr1p might bind this 3′UTR element to regulate the stability of its mRNAs. We thus investigated the ability of Rtr1p to associate with *RTR1* 3′UTR-containing mRNPs by testing the association of an N-terminally tagged 3X-Flag-Rtr1p with the *GFP-RTR1*_3′*UTR*_ mRNA in vivo using RNA immunoprecipitation (RIP). Because the Rtr1p phosphatase associates with the large subunit of RNA polymerase II, we controlled for the possibility that Rtr1p may associate with any mRNAs in complex with RNAPII at the site of transcription by also determining the association of Flag-Rtr1p with a *GFP* mRNA containing the *TEF1* 3′UTR, or the *RTR1* 3′UTR lacking the 3′UTR binding site (ΔBS). As determined by RT-qPCR (see Materials and Methods), Flag-tagged Rtr1p showed an approximately eightfold increase in association with *GFP-RTR1*_3′*UTR*_ over the “no-tag” control and a two- to threefold increase in association over the *GFP-TEF1*_3′*UTR*_ or *GFP-RTR1*_3′*UTR*, Δ*BS*_ (Fig. 3A). We thus conclude from these results that Rtr1p autoregulates its mRNA abundance through physical association with the *RTR1* mRNP, which is strongly dependent on the presence of the 15 nucleotide (nt) binding site in the 3′UTR. Based on these results we cannot conclude whether Rtr1p binds directly to this RNA element or whether this binding is mediated by other proteins within the *RTR1* mRNP.

### Dhh1p interacts with Rtr1p and facilitates decay of *RTR1* mRNAs

We gained insight into potential degradation factors that may participate in the Rtr1p-mediated decay of its own mRNAs through a previous study that performed mass-spectrometry analysis of proteins associated with Rtr1p (Smith-Kinnaman et al. 2014). In that study, Rtr1p was found to associate with the DExD/H-box helicase, Dhh1p. Additionally, tethering Dhh1p to various mRNAs has been shown to result in an increase in their turnover and also decreased protein levels (Carroll et al. 2011). Based on these observations, we hypothesized that Dhh1p could facilitate degradation of the *RTR1* mRNA through its interaction with Rtr1p. Indeed, we detected the association of Rtr1p with Dhh1p by coimmunoprecipitation using tagged strains (3X-Flag-RTR1 and HA-tagged Dhh1p) (Fig. 3B). Furthermore, we show that Rtr1p interacts with Dhh1p independently of any RNAs that may link the association since its association with Dhh1p was unaffected by RNase A treatment (Fig. 3C).

**FIGURE 4.**
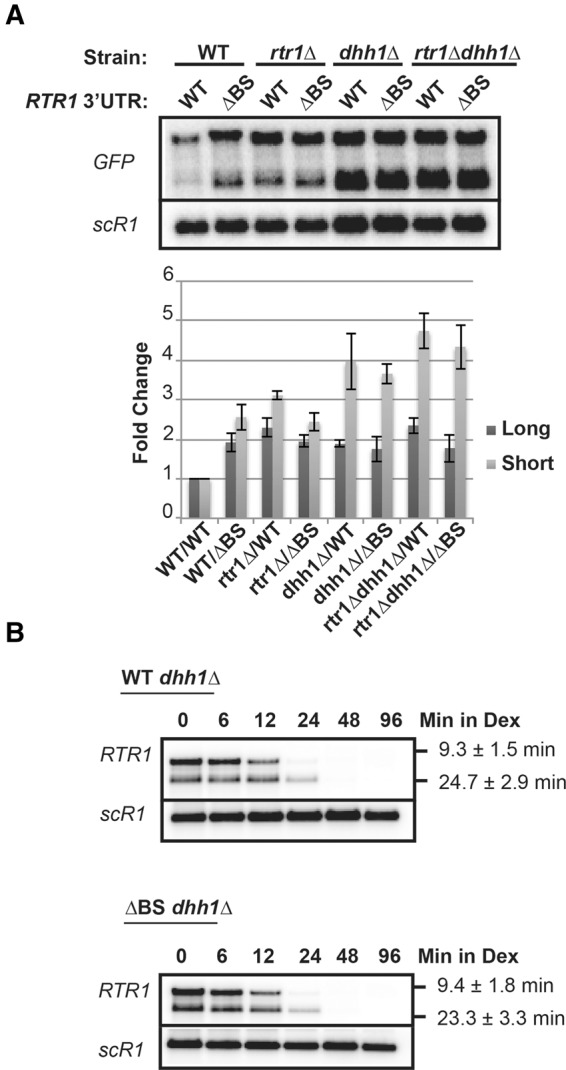
The impact of *DHH1* deletion on *RTR1* 3′UTR-containing mRNAs. (*A*) Northern blot analysis of steady-state WT or ΔBS GFP-RTR1_3′UTR/TER_ mRNAs in the WT, *rtr1*Δ, *dhh1*Δ, or *rtr1Δdhh1*Δ background. (*B*) Transcription shut-off assay with the galactose-driven promoter. DHH1 was knocked out in either the GAL-RTR1 WT or GAL-RTR1 ΔBS strains. Post-transcriptional stability was determined by harvesting the samples at the indicated time points after shifting from 2% galactose to 4% dextrose. Calculated half-lives are the result of three independent biological replicates.

To further demonstrate the impact of Dhh1p on *RTR1* decay, we analyzed the effect of Dhh1p absence on *RTR1* mRNA levels. Deletion of *DHH1* resulted in an increase in the *RTR1* 3′UTR mRNA levels; this increased accumulation was likely due to a combined effect of Dhh1p's general role in mRNA decay and also due to its role in the Rtr1-dependent degradation pathway (Fig. 4A). Dhh1p inactivation had a larger impact than the binding site or *RTR1* deletion alone, but deletion of the binding site had no cumulative effect in the *dhh1*Δ background (Fig. 4A). In addition, deleting *RTR1* in the *dhh1*Δ strain did not result in a further increase in accumulation of either the WT *RTR1* 3′UTR mRNAs or the ΔBS mRNAs. These epistatic results provide genetic evidence that Dhh1p functions in Rtr1p-mediated decay of the *RTR1* mRNA. Additionally, a transcription shut-off assay with the Gal system in the *dhh1*Δ strain demonstrates similar half-lives for the decay of the WT and ΔBS *RTR1* mRNAs, showing that deletion of the binding site element does not further increase *RTR1* stability in the absence of Dhh1p (Fig. 4B). Taken together, these data demonstrate that Dhh1p is involved in the Rtr1p-dependent turnover pathway of *RTR1* mRNAs.

### The Rtr1p binding element is required for degradation of *RTR1* mRNAs by Rex2p and Rex3p

**FIGURE 5.**
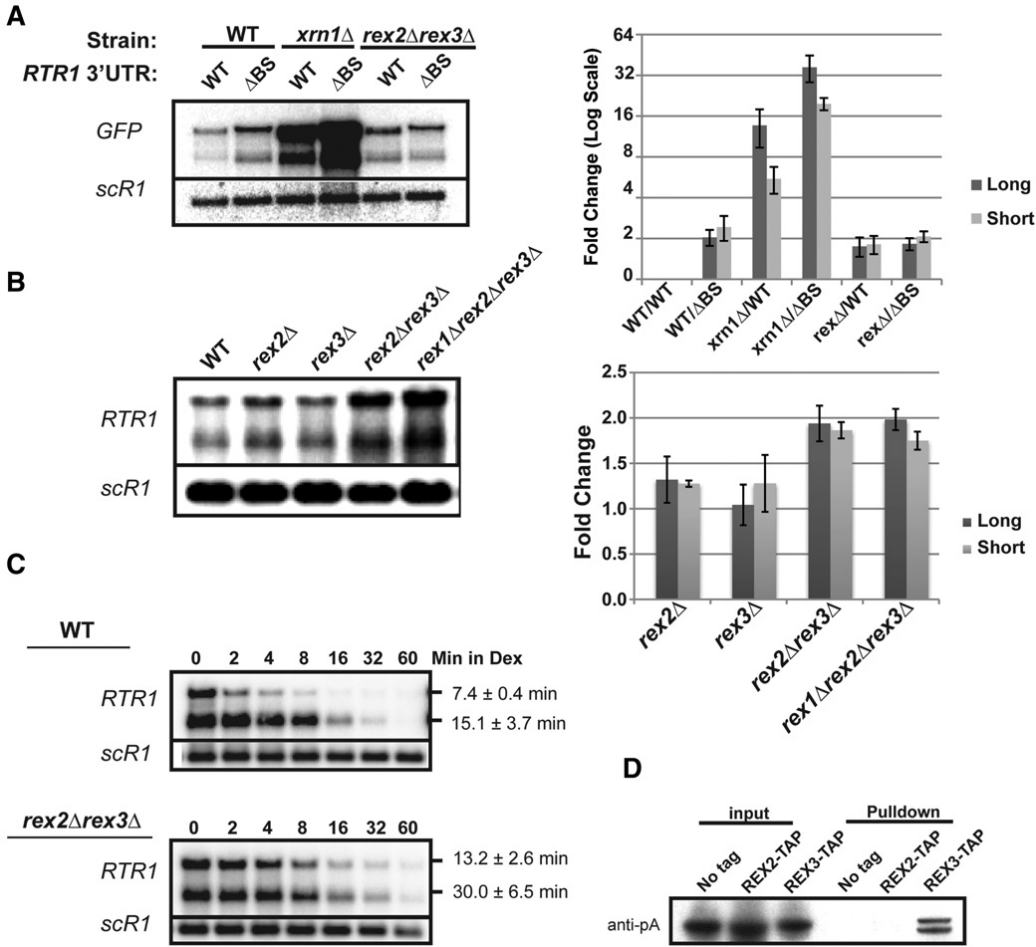
Testing the involvement of REX2 and REX3 in the Rtr1p autoregulation and degradation pathway by genetic and biochemical assays. (*A*) Northern blot analysis of steady-state WT or ΔBS GFP-RTR1_3′UTR/TER_ mRNAs in the WT, *xrn1*Δ, or *rex2Δrex3*Δ strains. Cultures were grown in SD-TRP and harvested during the log phase at OD 0.4–0.5. The quantitation for three independent biological replicates for this experiment is shown to the *right*. (*B*) Northern blot analysis of steady-state *RTR1* mRNA levels. Cultures were grown in YPD and harvested during the log phase at OD 0.4–0.5. The quantitation for three independent biological replicates for this experiment is shown to the *right*. (*C*) Post-transcriptional stability analysis of *RTR1* mRNAs expressed from the GAL1 promoter in either the WT or *rex2Δrex3*Δ background. Cultures were shifted from 2% galactose to 4% dextrose to turn off transcription from the GAL1 promoter. Calculated half-lives are the result of three independent biological replicates. (*D*) Pull-down assay performed to test the physical in vivo association of Rex2-TAP or Rex3-TAP with the tagged Dhh1p expressed from the BG1805 vector. Rex2-TAP, Rex3-TAP, or a “No tag” control was pulled down with calmodulin beads and tagged Dhh1p was detected with an anti-proteinA antibody.

To determine the downstream factors that are responsible for degrading the *RTR1* mRNAs through the 3′UTR binding site, we tested several exonuclease mutant strains. Inactivating exonucleases specifically involved in Rtr1p-mediated decay of *RTR1* should result in an increase in steady-state abundance of the WT *GFP-RTR1*_3′*UTR*_ mRNA but not the *GFP-RTR1*_3′*UTR*, Δ*BS*_ mRNA. We found that deletion of the *XRN1* gene coding for the major cytoplasmic 5′–3′ exonuclease Xrn1p resulted in a large synergetic increase in steady-state abundance when combined with the deletion of the binding site (Fig. 5A). These results showed that the Rtr1p-dependent degradation pathway is not epistatic to the deletion of *XRN1* and that another exonuclease is responsible for the Rtr1p-mediated turnover pathway. Because the steady-state abundance of endogenous *RTR1* mRNAs does not increase in either deletions of the nuclear exosome component, Rrp6p, or the cytoplasmic exosome component, Ski2p (Fig. 1A), we tested other exonucleases and focused on the Rex family of exonucleases. Implicated in the processing of 3′ ends of noncoding RNAs and having purported 3′–5′ exonuclease activity (van Hoof et al. 2000), we postulated that the Rex factors could also participate in the degradation of mRNAs. Deletion of *REX2* in combination with *REX3* resulted in an increased abundance of the *GFP-RTR1*_3′*UTR*_ (Fig. 5A) and of the endogenous *RTR1* mRNAs (Fig. 5A), while a triple deletion mutant, *rex1Δ rex2Δ rex3*Δ, did not exhibit further accumulation (Fig. 5B). Furthermore, the *GFP-RTR1*_3′*UTR*_ and *GFP-RTR1*_3′*UTR*, Δ*BS*_ mRNAs accumulated to the same degree in the *rex2Δ rex3*Δ strain, indicating that Rex2p and Rex3p are most likely responsible for the degradation of Rtr1p-targeted mRNAs through their 3′UTR. To ensure that the effects detected in the rex mutants were due to turnover defects, we demonstrated an increase in the half-life of the *RTR1* mRNAs in the *rex2Δ rex3*Δ strain as determined by a transcription shut-off assay with the Gal system controlling transcription of the endogenous *RTR1* gene (Fig. 5C). Overall these results demonstrate that the Rex2p and Rex3p proteins contribute to the degradation of *RTR1* mRNAs through a pathway dependent on the presence of the 3′UTR binding site recognized by Rtr1p.

We also analyzed the interaction of Dhh1p with the Rex2 and Rex3 exonucleases, which facilitate the degradation of *RTR1* mRNAs. We utilized TAP-tagged *REX2* and *REX3* strains to perform Calmodulin Binding Protein (CBP) pull-down of these proteins using calmodulin beads, and tested for the coprecipitation of proteinA-tagged Dhh1p. This pull-down assay showed that Dhh1p appears to interact solely with Rex3p and not Rex2p in “wild-type” cells (Fig. 5D). This result was somewhat unexpected considering the previous results, which implicated an overlapping function of Rex2p and Rex3p in the degradation of *RTR1*. However, this result suggests that the main degradation factor interacting with Dhh1p might be Rex3p. We note that Dhh1p migrates as a doublet in the pull-down lane. This could be due to a post-translationally modified form of Dhh1 that is enriched in the pull down. Alternatively, it is possible that the tagged version of Dhh1p is partially degraded by proteolysis during the pull-down experiment.

### Rtr1p-mediated decay potentially targets multiple classes of RNAs

**FIGURE 6.**
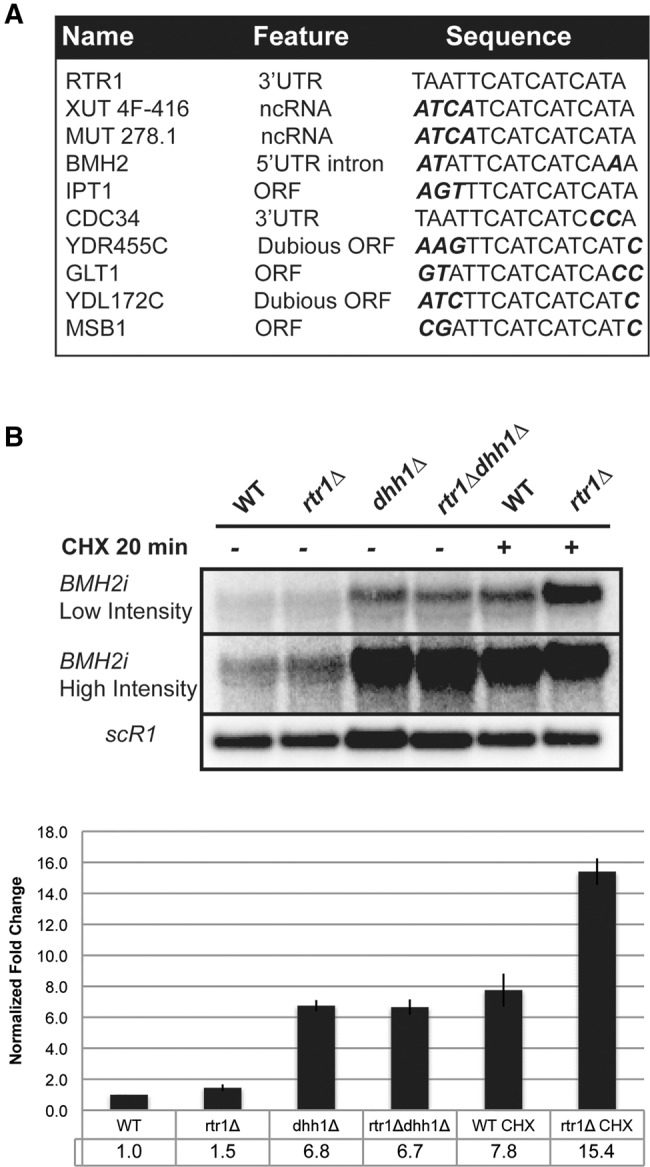
Additional potential targets of Rtr1p-mediated decay. (*A*) Hits for the BLAST search results for the *RTR1* 3′UTR *cis* element found within transcribed regions. Residues in the sequence that deviate from the *RTR1* element are bolded and italicized. (*B*) Northern blot analysis of steady-state *BMH2* pre-mRNAs in the *trans* mutants. An in vitro transcribed ^32^P-radiolabeled riboprobe antisense to the *BMH2* 5′UTR intron was hybridized to the membrane. All samples are derived from cultures grown in YPD and harvested at the log phase, OD 0.4–0.5. WT; *rtr1*Δ cultures were also treated with 100 μg/mL CHX for 20 min and then harvested. The intensity of the autoradiogram was adjusted within the Bio-Rad FX Quantity One software. The chart *below* shows the quantitation of two independent biological replicates.

We hypothesized that Rtr1p may play a role in the degradation of other cellular RNAs and performed a blast search of the 3′UTR *cis* element found in the *RTR1* 3′UTR (Fig. 6A). This search identified potential Rtr1p binding sites in RNAs expressed from a variety of genetic loci (ORFs, UTRs, and ncRNAs) (Fig. 6A). Of the various RNAs showing a sequence resembling the Rtr1p binding site, we tested the *BMH2* 5′UTR intron for accumulation of steady-state levels in the *rtr1*Δ, *dhh1*Δ, *rtr1Δdhh1*Δ strains by Northern blotting with a probe specific for the intron-containing mRNA and by real-time reverse transcriptase PCR (RT-qPCR). Our results show a moderate (∼1.5 fold) increase of the *BMH2* pre-mRNA in the *rtr1*Δ strain compared to the WT. Strikingly, there was no increase in the steady-state level in the *rtr1Δdhh1*Δ double mutant compared to the single *dhh1*Δ mutant (Fig. 6B). Previous tiling array and RNA-seq data show that the *BMH2* pre-mRNA is also an NMD target (Sayani et al. 2008; Kawashima et al. 2009), suggesting that Rtr1p-mediated decay of the BMH2 unspliced pre-mRNA may cooperate with NMD to degrade these unspliced transcripts. Indeed, a much larger increase in unspliced *BMH2* was detected in the *rtr1*Δ mutant compared to WT when NMD was inhibited by the translation elongation inhibitor, cyclohexamide (CHX) (Fig. 6B). This demonstrates that the impact of Rtr1p on the *BMH2* pre-mRNA is greater when the impact of NMD degradation on the unspliced species is diminished by translational inhibition. These results also show that Rtr1p-mediated decay may impact a larger number of transcripts than the Rtr1p transcripts.

## DISCUSSION

**FIGURE 7.**
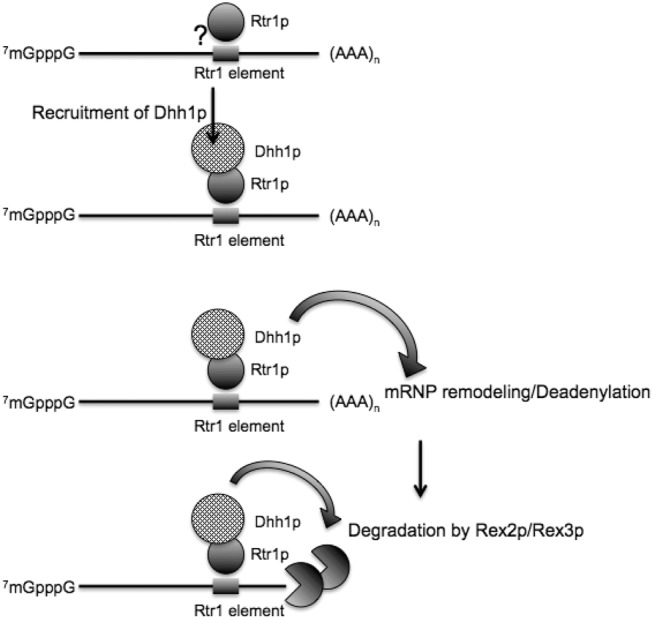
Model for Rtr1p-mediated mRNA decay. Depicted in the illustration is the proposed role of each factor involved in the pathway. An mRNA with the RBP binding element recruits the binding of Rtr1p to the mRNP. This event may occur cotranscriptionally given Rtr1p's association with the RNAP II CTD tail. Rtr1p physically associates with Dhh1p, which then may engage other degradation factors or serve to remodel the mRNP priming it for degradation. Dhh1p may also recruit deadenylases and ultimately, Rex3p. Rex3p would then digest the mRNA from the 3′ end.

In this study we report a novel role for the RNA Pol II CTD Ser5 and Tyr1 phosphatase Rtr1p in an mRNA degradation pathway that autoregulates its own mRNA and might also regulate a specific class of cellular transcripts. This pathway depends on the recognition of a *cis* element by Rtr1p, utilizes the Rex2p/Rex3p factors for degradation, and potentially involves the 5′–3′ DExD/H-box RNA helicase, Dhh1p, in promoting mRNA degradation through interaction with Rtr1p and the Rex proteins. Using a reporter system, we have demonstrated that the deletion of *RTR1* directly affects the degradation of a *RTR1* 3′UTR-containing mRNA. In our model, the binding of Rtr1p to the *RTR1* mRNP complex acts as a scaffold for the assembly of other mRNA degradation factors (Fig. 7). We propose that the interaction of Rtr1p with Dhh1p occurs upstream of deadenylation. The interaction with Dhh1p at this stage may serve to remodel the mRNP complex to prime it for degradation. Subsequently, the interaction of Rex3p with Dhh1p could potentially serve to recruit the exonuclease to degrade the mRNA. Thereby, the binding of Rtr1p to *RTR1* mRNP controls the overall expression by targeting a portion of the *RTR1* mRNA population for degradation in response to increasing Rtr1p protein levels. Given Rtr1p's localization to the site of transcription, an attractive hypothesis may be that Rtr1p is deposited onto the mRNP cotranscriptionally and may then potentially target the mRNA for degradation. As the binding site for Rtr1p is located in the 3′UTR region, Rtr1p may then get imprinted onto the transcript, thus altering its post-transcriptional stability. An overabundance of Rtr1p near the site of transcription would lead to a reduction of RTR1 mRNA through the downstream degradation pathway. In other cases, this may serve as an efficient quality-control mechanism to mark unspliced mRNAs like the *BMH2* pre-mRNA for degradation.

We have shown that Rtr1p associates with the mRNP complex of this mRNA and negatively regulates its stability. Though the in vivo association of Rtr1p with its own 3′UTR is clear, we have not resolved whether the binding of Rtr1p to the mRNP is due to a direct interaction with the binding element or whether the interaction takes place through the aid of an unidentified RBP. While Rtr1p does not have homology with any known RNA binding domain and an x-ray crystal structure of Rtr1p also did not necessarily reveal an RNA binding domain, it may be that the binding of Rtr1p to its target sequence, if direct, may occur in a noncanonical fashion and possibly through a disordered region (Hsu et al. 2014). Recent evidence suggests that a large population of previously unrecognized RBPs exist among metabolic enzymes and other factors not specifically recognized as being involved in RNA metabolism (Scherrer et al. 2010; Tsvetanova et al. 2010). Utilizing two approaches to UV crosslinking of RBPs to RNA, over 300 new RBPs have been discovered in HeLa cells, many of which are involved in metabolic processes (Castello et al. 2012). Unusually, these proteins may interact with RNA through repetitive and disordered regions or other nonclassical domain architectures (Castello et al. 2012; Neelamraju et al. 2015). Further, analysis of RIP-ChIP data sets has revealed that up to a third of known RBPs may post-transcriptionally autoregulate their own mRNAs including *PUF1*, *PUF2*, *PUF3*, and *PUF4* (Janga and Mittal 2011). This suggests that in addition to regulating other mRNA targets, autoregulatory feedback loops may be a common way for proteins to regulate their own intracellular concentrations. Rtr1p may fall into this category as well.

Whether direct or indirect, the binding of Rtr1p to the mRNP complex containing the 3′UTR and binding site is of significance to the regulation of the *RTR1* mRNA stability. This Rtr1p-mediated decay pathway, intriguingly, does not involve the well-characterized 5′–3′ cytoplasmic decay pathway or 3′–5′ decay by the cytoplasmic or nuclear exosome. Rather, we find that the degradation of *RTR1* by the Rtr1p-mediated decay pathway requires Rex2p and Rex3p. Known for their role in trimming the 3′ ends of noncoding RNAs, we find a novel role for these purported exonucleases in the degradation of mRNA. The fact that Dhh1p interacts only with Rex3p in our pull-down assay suggests that Rex3p might be the predominant exonuclease acting in this pathway. Thus it is possible that Rex2p only degrades the *RTR1* mRNA in the absence of Rex3p.

In this study we observe that Rtr1p-mediated decay of *RTR1* mRNA requires *DHH1*. The deletion of *DHH1* was epistatic to the deletion of both the binding site and *RTR1* itself, providing genetic evidence that Dhh1p functions in Rtr1p-mediated decay independently from its general role in mRNA degradation. Here, and in a previous study (Smith-Kinnaman et al. 2014), it has been determined that Rtr1p interacts with Dhh1p providing additional biochemical evidence that Dhh1p is involved in this decay pathway. Given that Dhh1p interacts with members of the Ccr4-Not deadenylase complex, a plausible role for Dhh1p's involvement would be to stimulate deadenylation of the *RTR1* mRNA prior to degradation by Rex2p/Rex3p (Coller et al. 2001; Fischer and Weis 2002; Maillet and Collart 2002). On the other hand, previous studies based on a *PGK1* reporter mRNA have concluded that Dhh1p acts downstream from deadenylation to stimulate decapping (Fischer and Weis 2002). Another study found that the enhanced degradation of an mRNA tethered to Dhh1p was independent of *CCR4* but not *XRN1* or other factors involved in 5′–3′ decay (Carroll et al. 2011). Other evidence suggests that the greatest amount of impairment in deadenylation results from deletion of both *CCR4* and *PAN2* and thus it is also possible that deadenylation may still occur in the absence of *CCR4* by the *PAN2*-*PAN3* deadenylases (Tucker et al. 2002; for review, see Wahle and Winkler 2013). The involvement of Dhh1p in Rtr1p-mediated decay, however, may involve a distinct mechanism, since normally, the action of Dhh1p in the degradation of mRNAs requires the 5′–3′ decay machinery which we have shown is not involved in Rtr1p-mediated decay. Rtr1p-mediated decay is also distinct from the previously established decay pathway involving Rbp1p (Nrg1p), which degrades the *POR1* mRNA and also interacts with Dhh1p, since a deletion of *RBP1* (*NRG1*) does not affect *RTR1* mRNA levels (Supplemental Fig. 1B; Chang and Lee 2012). Dhh1p may alternatively stimulate the decay of the *RTR1* mRNA through its interaction with Rex3p. Deadenylation may take place prior to this step and may be activated by another mechanism; though, we have not formally ruled out the possibility that Rex3p may digest the poly(A) tail and degrade the full-length mRNA.

In summary, Rtr1p-mediated decay is a novel mRNA degradation pathway that utilizes noncanonical exonucleases for degradation and may contribute to the stability and quality control of diverse RNAs, since the Rtr1p binding element was potentially found in a variety of cellular RNAs. Since the precise sequence determinants for Rtr1p binding are not fully understood, the list presented in Figure 6A might correspond only to a small subset of the actual population of RNAs targeted by Rtr1p-mediated decay.

## MATERIALS AND METHODS

### Plasmid and strain construction

All strains used in this study are listed in Supplemental Table 1. All oligonucleotides utilized for plasmid and strain construction are listed in Supplemental Table 2. Strains were constructed using standard PCR-based homologous recombination in yeast as described in the Geitz laboratory website (http://home.cc.umanitoba.ca/~gietz/). Single gene knockouts or promoter replacements were done with cassettes amplified from the pFA6a-kanMX6 or pFA6a-kanMX6-PGal1 (Longtine et al. 1998). The *rtr1*Δ was made using the CORE cassette (Storici and Resnick 2006). The *rex2*Δ, *rex3*Δ, *rex2Δrex3*Δ, and *rex1Δrex2Δrex3*Δ strains were constructed using the delitto perfetto approach (Storici and Resnick 2006). The 3X-Flag-RTR1 strain was constructed by inserting the CORE cassette in between the ATG and second codon of the *RTR1* ORF. The CORE cassette was excised with complementary IROs containing the 3X-Flag sequence and sequences homologous to the region flanking the CORE insertion site. The 3′ ends of the complementary IROs were extended using the Phusion High Fidelity DNA polymerase (New England BioLabs). The RTR1-3′UTR-ΔBS strain was generated by the delitto perfetto method as well (Storici and Resnick 2006).

The pRS404-GFP-RTR1_3′UTR/TER_ was made using the pRS404-PTEF-AGO1 plasmid purchased from Addgene. First, the GFP ORF was amplified from pFA6a-GFP(S65T)-HIS3MX6 plasmid with oligonucleotides that have 40 nt 5′ overhangs homologous to the regions flanking the *RTR1* ORF. This PCR product was transformed into the *rtr1*::CORE strain by the delitto perfetto approach (Storici and Resnick 2006). The gDNA from this strain was used to amplify the *RTR1* ORF, 3′UTR, and Terminator PCR product that was inserted into the SpeI/MluI sites of pRS404-PTEF-AGO1. The pRS404-GFP-TEF1_3′UTR/TER_ was constructed by amplifying the GFP ORF from pFA6a-GFP(S65T)-HIS3MX6 and inserting the PCR product into the SpeI/XhoI sites of pRS404-PTEF-AGO1.

### RNA extraction and Northern blotting

All RNA extractions and Northern blots were performed as described previously (Sayani and Chanfreau 2012). Oligonucleotides used to generate riboprobes are listed in Supplemental Table 2. The *scR1* ncRNA was probed for using the listed oligonucleotide, which was incubated with T4 PNK (New England BioLabs) and [γ-^32^P]-ATP (PerkinElmer) prior to hybridizing to the membranes.

### 3′ RACE and sequencing

The 3′ RLM-RACE kit (Thermo Fisher Scientific) was used for determining the 3′ ends of *RTR1* mRNAs. The custom forward primer contained a BamHI site along with the provided reverse anchor primer. The 3′ RACE products were ligated into the BamHI site in the pUG35 plasmid and transformed into competent DH5-α *E. coli* for sequencing.

### Transcription shut-off assays

Transcription was inhibited by the transcription inhibitor, Thiolutin (Enzo Life Sciences), at a final concentration of 3 μg/mL as described previously (Pelechano and Pérez-Ortín 2008). We tested 3, 6, 10, and 18 μg/mL Thiolutin and saw little difference on the *RTR1* half-lives. Samples were harvested by centrifugation at 3000 RPM for 1.5 min and transferred to 2 mL screw-cap tubes for RNA extraction. Samples were flash frozen in N_2_ (l) and stored in −20°C prior to RNA extraction.

For measuring half-lives of the RTR1 mRNAs with the GAL-RTR1 strains, overnight cultures were grown in YPGAL and back-diluted the next day to OD 0.05. When the cultures reached OD 0.4–0.5, the cells were spun down and resuspended in 20 mL YP media lacking sugar. A 2 mL zero minute time point was taken just prior to shifting the culture to 4% dextrose. To begin transcription shutoff at the GAL promoter, the remaining 18 mL of YP culture was added to a flask containing 3.6 mL 20% dextrose. Time points were taken by centrifuging 2 mL samples in screw-cap tubes, removing the supernatant, and flash freezing the cells in N_2_ (l). This protocol was adapted from the Coller laboratory protocol book (http:// www.case.edu/med/coller/Coller%20Protocol%20Book.pdf).

### RNA immunoprecipitation and RT-qPCR

Overnight cultures for each sample were back diluted to 0.05 and then grown to OD 0.5. When the cultures reached OD 0.5, 20 OD units were harvested from each culture (∼40 mL). The samples were pelleted then washed with 10 mL cold ddH_2_O. Pellets were spun down and then resuspended in 1 mL cold ddH_2_O and transferred to a 2 mL Eppendorf tube. The supernatant was removed and the pellet was frozen at −80°C.

The pellets were thawed and resuspended in 600 μL NET-2 Buffer (40 mM Tris–HCl pH 7.5, 150 mM NaCl, and 0.05% IGEPAL). Twelve microliters of 50× protease inhibitor cocktail was added (Roche Diagnostics, F. Hoffman-La Roche Ltd.) along with glass beads. The tubes were then vortexed five times at 4°C for 45 sec each time with 45-sec intervals on ice between each vortexing. The Eppendorf tubes were then pierced with a 23G flamed needle at the bottom of the tube and placed into a 2 mL screw cap tube. The tubes were taped together and spun down for 1 min at 3000 rpm to allow the lysate to flow from the top to the bottom tube. Samples were then centrifuged for 20 min at 4°C at maximum speed to pellet the insoluble fraction. The supernatant was transferred to a clean Eppendorf tube and the total protein/RNA was then quantitated by Nanodrop (Thermo Fisher Scientific). Of note, 2.5 OD units of each sample were then used for immunoprecipitation and 2.5 OD units was also used for the input by directly extracted with phenol/chloroform/isoamyl alcohol (PCA). To prepare the input total RNA, the volume of each sample was raised to 400 μL with NET-2 buffer and 40 μL 3 M sodium acetate and 5 μL 20% SDS were added. Four hundred microliters of PCA was added and vortexed for 1 min. The samples were spun for 3 min at maximum speed. The supernatant was transferred to a tube containing 1 mL 100% ethanol and 1 μL Glycoblue (Ambion). The samples were precipitated overnight at −80°C, spun at maximum speed for 10 min, and washed with 70% ethanol prior to resuspending in 15 μL RNase-free water (Ambion).

The immunoprecipitation was done by conjugating the Flag antibody (M2 monoclonal antibody from Sigma) to Protein G Sepharose beads (4 Fast Flow by GE Healthcare). The Protein G sepharose beads (20 μL per sample) were first washed with NET-2 buffer twice and resuspended to 400 μL with NET-2 buffer. Five microliters of Flag antibody per sample was added to the tube and the mixture was rotated for 1 h at 4°C. After conjugation, the beads were washed twice with NET-2 buffer and aliquoted in separate tubes for each sample. Four hundred microliters of 2.5 OD RNA/protein lysate for each sample was added to the aliquoted beads and the mixture was rotated for 1 h at 4°C. The beads were then washed four times with 1 mL cold NET-2 buffer each time and resuspended in 400 μL NET-2 buffer after the fourth wash. Four hundred microliters PCA, 40 μL 3 M sodium acetate, and 5 μL 20% SDS were then added directly to the beads/NET-2 buffer and the RNA was extracted the same as for the input RNA.

RNA was reverse transcribed with the Superscript III First-Strand synthesis kit (Life Technologies). The cDNAs were diluted 10-fold and 1 μL of each cDNA was used for qPCR. qPCR was performed using the GFP TaqMan assay and the TaqMan Universal Master Mix II, with UNG (Applied Biosystems). The qPCR runs were done on the Bio-Rad CFX Connect Realtime PCR detection system. The GFP TaqMan assay was validated using serial dilutions of the pFA6a-kanMX6-GFP plasmid across six orders of magnitude. The PCR efficiency was then calculated using the CFX Manager software to be 94.6%.

### Coimmunoprecipitation and pull-down assays

One-liter cultures of 3X-Flag-RTR1 or WT strains harboring the BG1805-DHH1 plasmid (Yeast ORF collection from Dharmacon) were grown in SGAL-URA and harvested at log phase, OD 0.6. The cultures were spun down and resuspended in 10 mL cold IPP150 buffer (10 mM TRIS–HCl pH8.0, 150 mM NaCl, and 0.1% IGEPAL). The cells were then dripped into ∼400 mL N_2_ (l) in a Nalgene beaker. After freezing the cell suspension, the samples were stored at −80°C. The cells were then mechanically lysed in N_2_ (l) using the Retsch MM400 with four cycles of shaking at 12 Hz for 3 min each. Between cycles, the capsule was incubated in N_2_ (l). The powdered cells were then transferred to centrifuge tubes, allowed to thaw on ice for ∼1 h, and spun at 12,000 RPM (JA 25.50 rotor) for 10 min with the Beckman-Coulter centrifuge set at −8°C. After the insoluble fraction was pelleted, the supernatant was transferred to 15 mL falcon tubes and protease inhibitor was added to 1× concentration. Five hundred microliters of aliquots from each sample were precipitated with TCA for the input. Anti-Flag conjugated to Protein G sepharose beads were added to the supernatant samples and the complexes were precipitated at 4°C overnight in the presence of Protease 3C. The next day, the beads were washed four times with cold IPP150 buffer. The beads were transferred after the last wash to a clean Eppendorf tube and boiled in Thorner buffer (40 mM TRIS pH 8, 5% SDS, 8 M urea, 100 μM EDTA). Western blotting was carried out using an HA antibody.

REX2-TAP and REX3-TAP strains were purchased from GE Dharmacon. These strains or the wt strain were transformed with the BG1805-DHH1 vector. One-liter cultures were grown in SGAL-URA and harvested at log phase, OD 0.6. The calmodulin pull-down assay was performed the same as the Anti-Flag co-IP, except the lysate was applied to Calmodulin Sepharose 4B beads (GE Healthcare) without antibodies. IPP150 Calmodulin binding buffer (10 mM β-mercaptoethanol, 10 mM TRIS–HCl pH8.0, 150 mM NaCl, 1 mM Mg-acetate, 1 mM imidazole, 2 mM CaCl_2_, and 0.1% IGEPAL) was used in lieu of IPP150 for the pulldown. Western blotting for these experiments was done using a proteinA antibody.

## SUPPLEMENTAL MATERIAL

Supplemental material is available for this article.

## Supplementary Material

Supplemental Material
