# Supplementary material for: The Rtr1p CTD phosphatase autoregulates its mRNA through a degradation pathway involving the REX exonucleases
Source: RNA. 2016 Apr;22(4):559–70. doi: 10.1261/rna.055723.115 (PMC4793211; doi:10.1261/rna.055723.115)
Supplement: Supplemental Material [file supp_22_4_559__index.html]

The Rtr1p CTD phosphatase autoregulates its mRNA through a degradation pathway involving the REX exonucleases — The Rtr1p CTD phosphatase autoregulates its mRNA through a degradation pathway involving the REX exonucleases — Supplemental Material 

# The Rtr1p CTD phosphatase autoregulates its mRNA through a degradation pathway involving the REX exonucleases

## Supplemental Material

**Files in this Data Supplement:**

- Supp Figure 1.tif
- Supp Material.docx
