# Supplementary figures and images for: The Rtr1p CTD phosphatase autoregulates its mRNA through a degradation pathway involving the REX exonucleases
Source: RNA. 2016 Apr;22(4):559–70. doi: 10.1261/rna.055723.115 (PMC4793211; doi:10.1261/rna.055723.115)

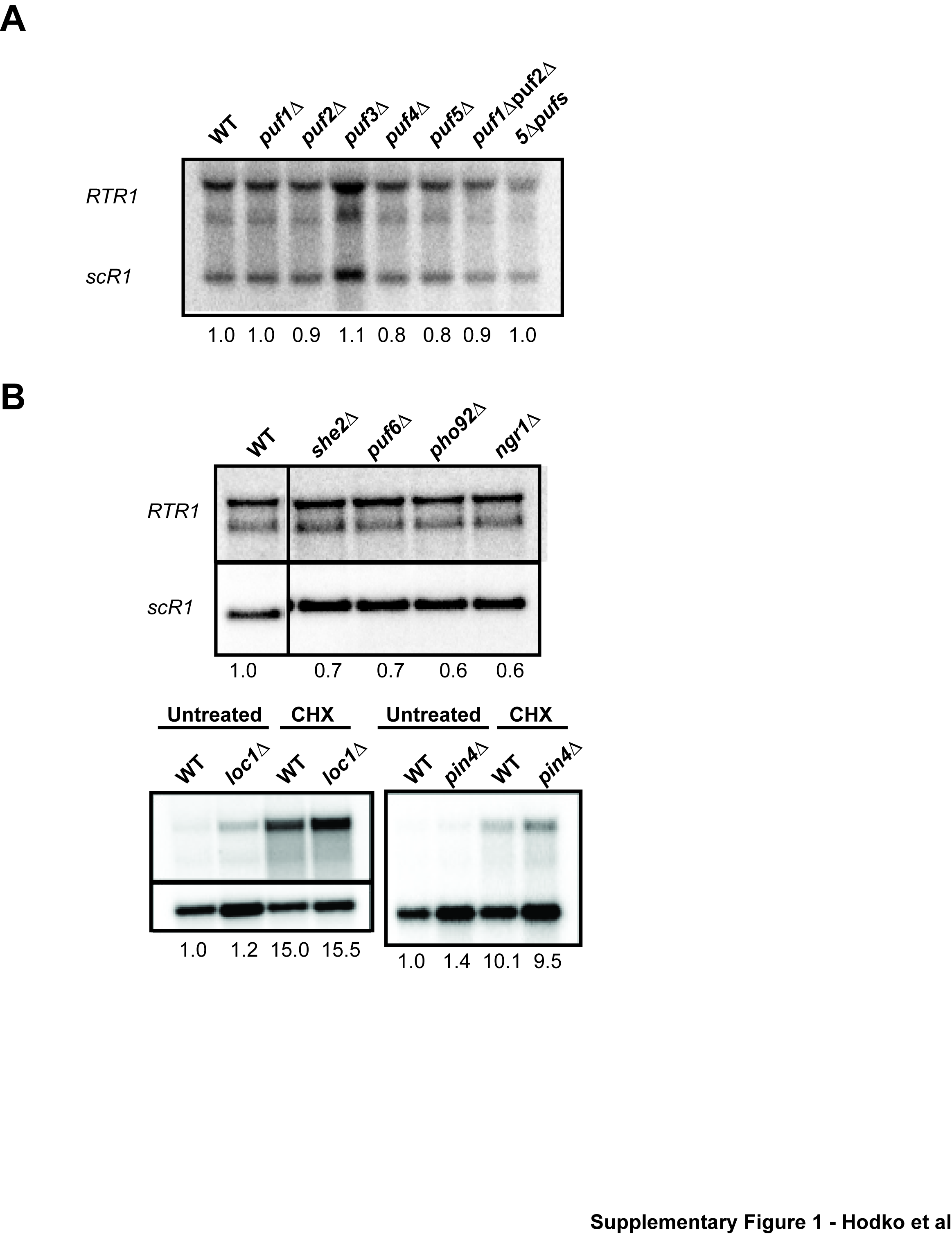

Supplement: Supplemental Material [file supp_055723.115_SuppFigure1.tif]
