# Supplementary material for: The Rtr1p CTD phosphatase autoregulates its mRNA through a degradation pathway involving the REX exonucleases
Source: RNA. 2016 Apr;22(4):559–70. doi: 10.1261/rna.055723.115 (PMC4793211; doi:10.1261/rna.055723.115)
Supplement: Supplemental Material [file supp_055723.115_SuppMaterial.docx]

**Supplementary figure 1.** Steady-state analysis of RBP deletion strains by northern blotting **(A)** *puf1∆*, *puf2∆*, *puf3∆*, *puf4∆*, *puf5∆*, *puf1∆ puf2∆,* or the *5∆pufs* (quintuple deletion mutant of PUFs 1-5) were grown along with the isogenic wildtype strain in YPD and harvested in log phase at OD 0.5. Northern blot analysis was carried out with the *RTR1* ORF riboprobe. **(B)** The experiment was carried out exactly as in (A), except that, *loc1∆* and *pin4∆* were also subjected to CHX treatment for 20 minutes. All lanes shown in the left panel were from the same northern blot, but irrelevant lanes in between WT and *she2∆* were omitted.

**Supplementary Table 1.** *S. cerevisiae* strains used in this study.

| **Background/Strain** | **Mutation** | **Source** |
| --- | --- | --- |
| **BMA64-a** | WT (derivative of W303) | Lab stock |
| **BMA64-a** | *upf1Δ* | Sayani and Chanfreau 2012 |
| **BMA64-a** | *rrp6Δ* | Sayani and Chanfreau 2012 |
| **BMA64-a** | *upf1Δrrp6Δ* | Sayani and Chanfreau 2012 |
| **BY4742** | WT (Open Biosystems) | Lab stock |
| **BMA64-a** | *rex2Δ* | This study |
| **BMA64-a** | *rex3Δ* | This study |
| **BMA64-a** | *rex2Δrex3Δ* | This study |
| **BMA64-a** | *rex1Δrex2Δrex3Δ* | This study |
| **yRP841** | MATα *leu2‐3,112, lys2, trp1‐1, ura3‐52, cup1::LEU2/PM* | Olivas and Parker 2000 |
| **yRP1243** | *puf1Δ* | Olivas and Parker 2000 |
| **yRP1237** | *puf2Δ* | Olivas and Parker 2000 |
| **yRP1241** | *puf3Δ* | Olivas and Parker 2000 |
| **yRP1245** | *puf4Δ* | Olivas and Parker 2000 |
| **yRP1239** | *puf5Δ* | Olivas and Parker 2000 |
| **yRP1290** | *puf1Δpuf2Δ* | Olivas and Parker 2000 |
| **yRP1253** | *5Δpufs* | Olivas and Parker 2000 |
| **BMA64-a** | *rtr1*::CORE | This study |
| **BMA64-a** | RTR1 N-term CORE insertion | This study |
| **BMA64-a** | 3X-FLAG-RTR1 | This study |
| **BMA64-a** | *rtr1*::GFP | This study |
| **BMA64-a** | *xrn1*::TRP | This study |
| **BMA64-a** | RTR1-3’UTR::CORE insertion | This study |
| **BMA64-a** | RTR1-3’UTR-ΔBS | This study |
| **BMA64-a** | *dhh1*::KAN^R^ | This study |
| **BMA64-a** | *rtr1::*CORE *dhh1*::KAN^R^ | This study |
| **BMA64-a** | HIS3MX6-PGAL1-RTR1 | This study |
| **BMA64-a** | RTR1-3’UTR-ΔBS HIS3MX6-PGAL1-RTR1 | This study |
| **BMA64-a** | RTR1-3’UTR-ΔBS HIS3MX6-PGAL1-RTR1 *dhh1*::KAN^R^ | This study |
| **BMA64-a** | rex2Δrex3Δ HIS3MX6-PGAL1-RTR1 | This study |
| **BMA64-a** | dhh1::KAN^R^ HIS3MX6-PGAL1-RTR1 | This study |
| **REX2-TAP** | S288C: (ATCC 201388: MATa his3Δ1 leu2Δ0 met15Δ0 ura3Δ0) | GE Dharmacon |
| **REX3-TAP** | S288C: (ATCC 201388: MATa his3Δ1 leu2Δ0 met15Δ0 ura3Δ0) | GE Dharmacon |
| **BY4742** | WT | GE Dharmacon |
| **BY4742** | *rrp6Δ* | GE Dharmacon |
| **BY4742** | *ski2Δ* | GE Dharmacon |
| **BY4742** | *she2Δ* | GE Dharmacon |
| **BY4742** | *puf6Δ* | GE Dharmacon |
| **BY4742** | *pho92Δ* | GE Dharmacon |
| **BY4742** | *ngr1Δ* (*rbp1Δ*) | GE Dharmacon |
| **BY4742** | *loc1Δ* | GE Dharmacon |
| **BY4742** | *pin4Δ* | GE Dharmacon |

**Supplementary Table 2.** Oligonucleotides used in this study

| Name | **Sequence** | **Purpose** |
| --- | --- | --- |
| GFP-F | TGAGTAAAGGAGAAGAACTTTTCAC | PCR template for Riboprobe |
| GFP-T3-R | GGCTAAATTAACCCTCACTAAAGGTTTGTATAGTTCATCCATGCCA | PCR template for Riboprobe |
| RTR1-F | GATATTAAGGAAACGGCGTTAATCC | PCR template for Riboprobe |
| RTR1-T3-R | GGCTAAATTAACCCTCACTAAAGGCTGAATTTAGGTGCATTGATATAGC | PCR template for Riboprobe |
| BMH2-791UP-F | AGCTCCTTCCACAACCACCTTCATC | PCR template for Riboprobe |
| BMH2-290UP-T3-R | GGCTAAATTAACCCTCACTAAAGGATAACATTTGCCCCTTTCGACCGAC | PCR template for Riboprobe |
| scR1 probe | ATCCCGGCCGCCTCCATCAC | Oligoprobe for scR1 |
| RTR1-3'RACE-BamHI | CGCCGCCGCGGATCCGACGCTGCAAGAAGAATCGTTCAC | 3’RACE |
| RTR1-UP-INS-CORE-F | CGGCATCTTAGTTTGAAAAATTAGGACGAAGTTAACAAGAATAAGAAATG  GAGCTCGTTTTCGACACTGG | Insertion of CORE at Rtr1 N-term |
| RTR1-UP-INS-CORE-R | TGCTTTTGGAAAGGGATTAACGCCGTTTCCTTAATATCTTCAATCGTCGC TCCTTACCATTAAGTTGATC | Insertion of CORE at Rtr1 N-term |
| GFP-Replace-RTR1-F | CATCGGCATCTTAGTTTGAAAAATTAGGACGAAGTTAACAAGAATAAGAAATGTCTTTAATTAACAGTAAAGGAG | Replacement of RTR1 ORF with GFP for downstream plasmid construction |
| GFP-Replace-RTR1-R | AATGAAACGTCCATAATCTGTTCTGAATTTAGGTGCATTGATATAGCCGGCTATTTGTATAGTTCATCCATGC | Replacement of RTR1 ORF with GFP for downstream plasmid construction |
| RTR1-UP-3xFLAG-F | GTTTGAAAAATTAGGACGAAGTTAACAAGAATAAGAAATGGACTACAAAGACCATGACGGTGATTATAAAGAT CATGACATCGAT | RTR1 N-term 3xFLAG insertion |
| RTR1-UP-3xFLAG-R | AAGGGATTAACGCCGTTTCCTTAATATCTTCAATCGTCGCCTTGTCATCGTCATCCTTGTAATCGATGTCATGATCTTTATAATC | RTR1 N-term 3xFLAG insertion |
| XRN1-F1 | ACTTGTAACAACAGCAGCAACAAATATATATCAGTACGGTCGGATCCCCGGGTTAATTAA | XRN1 knockout |
| XRN1-R1 | TAAAGTAACCTCGAATATACTTCGTTTTTAGTCGTATGTTGAATTCGAGCTCGTTTAAAC | XRN1 knockout |
| RTR1-GAL-F4 | GACACATTGAGGAGCGAATTGAACAATTCATAAACATTCCGAATTCGAGCTCGTTTAAAC | PGAL1 promoter replacement at RTR1 locus |
| RTR1-GAL-R2 | AAGGGATTAACGCCGTTTCCTTAATATCTTCAATCGTCGCCATTTTGAGATCCGGGTTTT | PGAL1 promoter replacement at RTR1 locus |
| DHH1-F1 | ATCCCAGGCCTAAAATACGACAAGAAAGAAAATAGTAGTA CGGATCCCCGGGTTAATTAA | DHH1 knockout |
| DHH1-R1 | GCGTATCTCACCACAGTAGTTATTTTTTCTTAGATATTCT GAATTCGAGCTCGTTTAAAC | DHH1 knockout |
| REX2-CORE-INS-F | AAATGAAAAAAAAGAAAAGGAGCTTTCACAAATAAACAGAAGAGATCAAG GAGCTCGTTTTCGACACTGG | REX2::CORE knockout |
| REX2-CORE-INS-R | TGTGAAATATTTGAAAAATTTCACTTCTTTTTTCTTTTTTTTTTTCTACA TCCTTACCATTAAGTTGATC | REX2::CORE knockout |
| REX2-CORE-EX1 | AAAGAAAAGGAGCTTTCACAAATAAACAGAAGAGATCAAGTGTAGAAAAAAAAAAAGAAAAAAGAAGTGAAATTTTTCAA | Excision of CORE from REX2 locus |
| REX2-CORE-EX2 | TTGAAAAATTTCACTTCTTTTTTCTTTTTTTTTTTCTACACTTGATCTCTTCTGTTTATTTGTGAAAGCTCCTTTTCTTT | Excision of CORE from REX2 locus |
| REX3-CORE-INS-F | GTGTTTCAGTATACATTCAGTTTGACTATATATCAAGAGAAAGCTTTAGTGAGCTCGTTTTCGACACTGG | REX3::CORE knockout |
| REX3-CORE-INS-R | TATGAAATGTAATAACTATATATGTCTGCTCAACTTTGAATATGATCACA TCCTTACCATTAAGTTGATC | REX3::CORE knockout |
| REX3-CORE-EX1 | ATACATTCAGTTTGACTATATATCAAGAGAAAGCTTTAGTTGTGATCATATTCAAAGTTGAGCAGACATATATAGTTATT | Excision of CORE from REX3 locus |
| REX3-CORE-EX2 | AATAACTATATATGTCTGCTCAACTTTGAATATGATCACAACTAAAGCTTTCTCTTGATATATAGTCAAACTGAATGTAT | Excision of CORE from REX3 locus |
| REX1-CORE-INS-F | ACCAAGGATGACTGAGGAAGAAAACAATAATAGACTATACTCAGGCAAACGAGCTCGTTTTCGACACTGG | REX3::CORE knockout |
| REX1-CORE-INS-R | ATATATATATATATATATATATATATATTTATATATTTATACACATAGAA TCCTTACCATTAAGTTGATC | REX3::CORE knockout |
| REX1-CORE-EX1 | ACTGAGGAAGAAAACAATAATAGACTATACTCAGGCAAACTTCTATGTGTATAAATATATAAATATATATATATATATAT | Excision of CORE from REX3 locus |
| REX1-CORE-EX2 | ATATATATATATATATATTTATATATTTATACACATAGAAGTTTGCCTGAGTATAGTCTATTATTGTTTTCTTCCTCAGT | Excision of CORE from REX3 locus |
